# Circadian Rhythms in Murine Ocular Tissues Including Sclera Are Affected by Neurobasal A Medium Preincubation and Mouse Strain, but Not Sex

**DOI:** 10.1167/iovs.66.6.62

**Published:** 2025-06-20

**Authors:** Nemanja Milićević, Cristina Sandu, Etienne Challet, Teemu O. Ihalainen, Soile Nymark, Marie-Paule Felder-Schmittbuhl

**Affiliations:** 1 Faculty of Medicine and Health Technology, Tampere University, Tampere, Finland; 2 Centre National de la Recherche Scientifique, Université de Strasbourg, Institut des Neurosciences Cellulaires et Intégratives, Strasbourg, France

**Keywords:** bioluminescence, circadian rhythm, retinal pigment epithelium (RPE), sex difference, retina, cornea

## Abstract

**Purpose:**

Our understanding of ocular clocks has been profoundly advanced by the development of real-time recording of bioluminescence of PER2-luciferase (PER2::LUC) knock-in mouse explants. However, the effect of sex, mouse strain, and culturing conditions on ocular clocks remains unknown. Here, we studied the role these variables play on PER2::LUC bioluminescence rhythms of ocular tissues: retinas, corneas, and posterior eye cups (PECs). We also tested the hypothesis that the sclera contains a circadian oscillator by using scraped PECs as a proxy.

**Methods:**

Retinas, corneas, and intact and scraped PECs were obtained from male and female PER2::LUC knock-in mice maintained on either a pigmented C57BL/6J or albino RjOrl:SWISS background. PER2::LUC bioluminescence rhythms in ocular tissues were measured using a Lumicycle.

**Results:**

We compared PER2::LUC bioluminescence rhythms between ocular tissues and found that all ocular tissues oscillated, including the scraped PECs, which were previously not known to oscillate. The rhythms in scraped PECs had lower amplitudes, longer periods, and distinct acrophases compared with other ocular tissues. Immunolabeling revealed the presence of the protein product of the clock gene BMAL1 in the sclera. Ocular tissues of RjOrl:SWISS mice oscillated with higher amplitudes compared with the ones of C57BL/6J, with corneal rhythms being most affected by mouse strain. A 24-hour preincubation with Neurobasal A medium affected rhythms of ocular tissues, whereas sex differences were not detected for these rhythms.

**Conclusions:**

We discovered a novel oscillator in the sclera. PER2::LUC bioluminescence rhythms in murine ocular tissues are affected by Neurobasal A medium preincubation mouse strain but not sex.

Rhythmic 24-hour changes in the Earth's environment are one of the most salient selection pressures for all living beings. These challenges were met by the development of circadian rhythms, that is, innate, 24-hour biological cycles that govern the physiological, behavioral, and biochemical processes of living organisms.^1^ In mammals, these rhythmic changes are orchestrated by the “central clock” located in the suprachiasmatic nucleus (SCN) in the brain, which, in turn, coordinates the timing of peripheral clocks.^2^ The core molecular components generating these oscillations are virtually the same in all cell types with transcription factors, such as PER1-2, CLOCK, BMAL1, CRY1-2, REV-ERBs, and RORs driving 24-hour periodicity of transcription-translation via negative interlocking feedback loops.^3^ The coordinated activity of these factors drives rhythmic expression of “clock-controlled genes,” thereby enabling rhythmic adaptations in physiological, behavioral, and biochemical processes.^3^

The eye is considered as a peripheral clock with unique properties: it lies in direct contact with the principal time-giver (*Zeitgeber*) – light, and it is independent from the central clock, the SCN.^4^^,^^5^ Our understanding of ocular clocks has been profoundly advanced by the development of real-time monitoring of bioluminescence of PER2-luciferase (PER2::LUC) knock-in mouse explants.^6^^,^^7^ Circadian rhythms were found in numerous ocular tissues, including the retina,^8^^–^^10^ the cornea,^6^^,^^11^ the posterior eye cup (PEC) as a proxy for the retinal pigment epithelium (RPE),^12^ and ciliary body.^13^^,^^14^ Substantial effort was made in elucidating the regulation of these clocks. For example, it was found that the phase of ocular clocks is set by secreted neurohormones and neurotransmitters, such as dopamine as a day signal and melatonin as a night signal.^4^^,^^13^ Each ocular clock is regulated in a distinct way, with dopamine being critical for entraining (i.e. setting the phase of) the retinal^9^ and PEC rhythms,^15^ whereas with melatonin for corneal rhythms.^11^^,^^16^

There is considerable variability in culturing protocols of PER2::LUC explants, with some groups using a 24-hour preincubation with Neurobasal A medium and continued culturing using other media,^9^^,^^17^^–^^19^ or without preincubation and culturing with M199 medium^7^ or DMEM medium.^6^^,^^16^^,^^20^ Little is known about the influence these conditions play in PER2::LUC bioluminescence recordings. Our understanding of the mouse strain effect on ocular clocks is also limited. Retinal explants of C57BL/6 and B6C3 mice show similar rhythms,^9^ whereas the regulation of corneal rhythms of C57BL/6 and C3Sn mice is distinct.^16^ To the best of our knowledge, there have been no studies on ocular clocks using mice with an albino background. Furthermore, the role of animal sex on ocular clocks has been largely ignored. Recent work showed no differences between male and female retinal rhythms.^21^ However, it is unknown whether sex affects the corneal or PEC rhythms.

In this paper, we studied PER2::LUC bioluminescence rhythms in various ocular tissues: corneas, retinas, PECs, and scraped PECs as a proxy for sclerae. We then tested the possibility that PER2::LUC mice on an albino RjOrl:SWISS background differ in bioluminescence rhythms in ocular tissues compared with pigmented C57BL/6J PER2::LUC mice. Finally, we explored the role that culturing conditions and sex differences play in PER2::LUC bioluminescence rhythms in ocular tissues.

## Materials and Methods

### Mice

**Figure 1.**
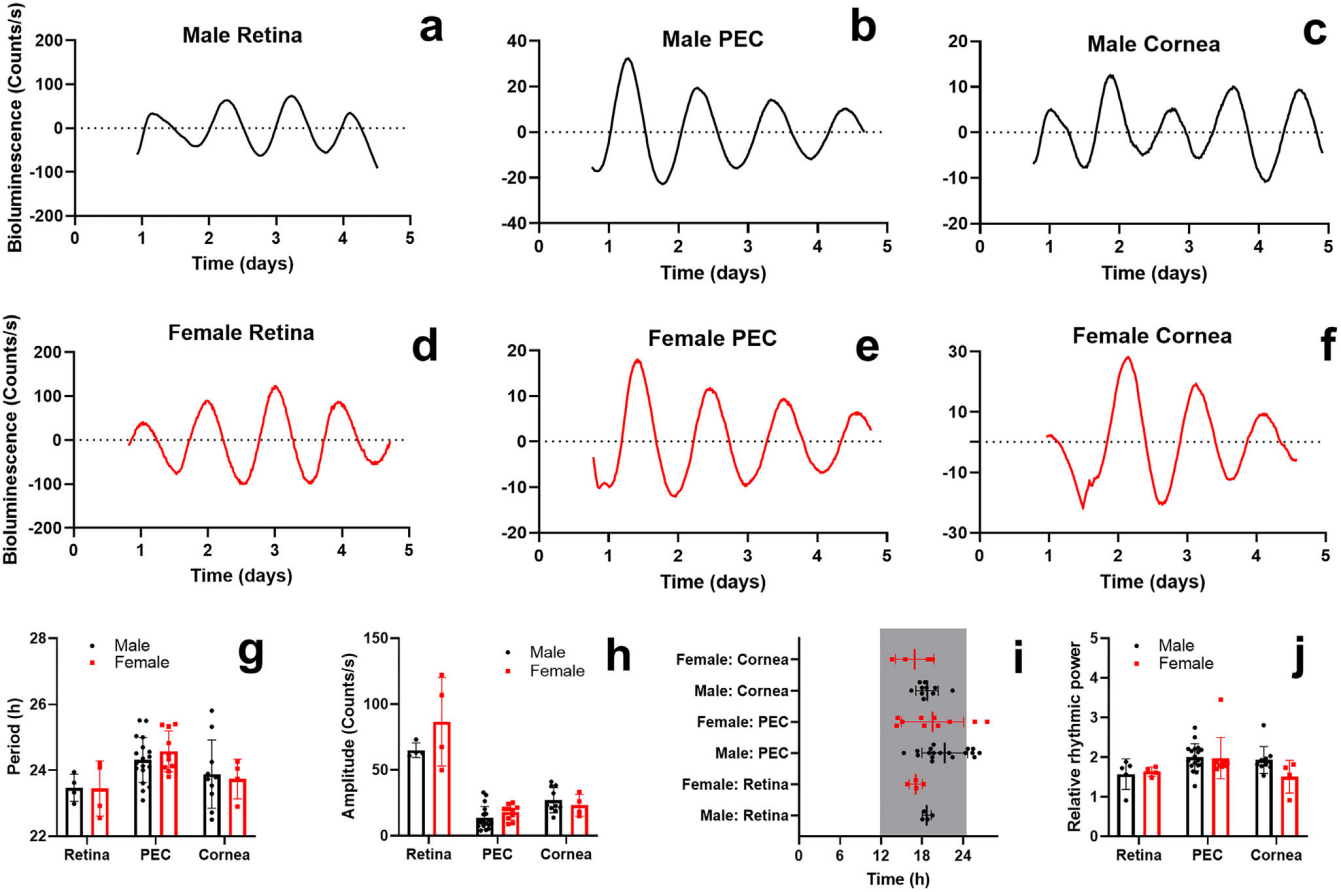
**Sex does not affect circadian rhythms of PER2::LUC bioluminescence of ex vivo ocular tissues**. Representative examples of bioluminescence rhythms in retina, PEC and cornea of (**a**–**c**) male and (**d**–**f**) female PER2::LUC mice bread on a C57BL/6J background. Animal sex did not significantly affect the following parameters of PER2::LUC bioluminescence rhythms: (**g**) period length, (**h**) amplitude, (**i**) acrophase, and (**j**) relative rhythmic power. The *arrows* indicate media change. Time in (**i**) is projected ZT with ZT12 = lights off. The *gray rectangle* represents subjective nighttime. Individual data points are plotted together with means ± SD. PEC, posterior eye cup. *N* = 4 to 18.

**Figure 2.**
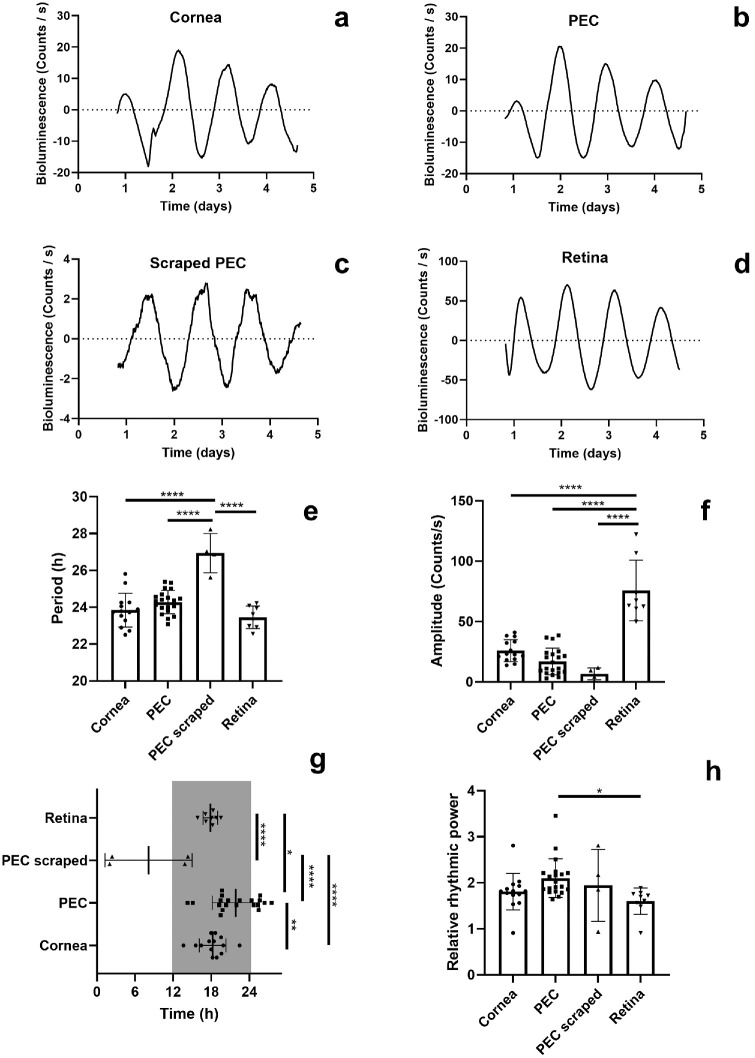
**Circadian rhythms of PER2::LUC bioluminescence in ex vivo ocular tissues**. Representative examples of PER2::LUC rhythms in (**a**) the cornea, (**b**) the intact PECs, (**c**) the scraped PEC, and (**d**) the retinas of mice bred on a C57BL/6J background. The bioluminescence rhythms of scraped PEC show (**e**) longer periods and (**f**) lower amplitudes compared with other ocular tissues. (**g**) The first obvious peak of PER2::LUC bioluminescence occurred earlier within the 24-hour cycle in scraped PECs compared with other ocular tissues. (**h**) The bioluminescence rhythms of PECs oscillated with higher relative rhythmic power compared with the retinas. Time in (**g**) is projected ZT with ZT12 = lights off. The *gray rectangle* represents subjective nighttime. Individual data points are plotted together with means ± SD. Holm-Sidak's post hoc test comparison, *P < 0.05, **P < 0.01, ****P < 0.0001. PEC, posterior eye cup. *N* = 4 to 20.

All experimental protocols were carried out according to the Association for Research in Vision and Ophthalmology Statement on Use of Animals in Ophthalmic and Vision Research, the European Parliament, and The Council of the European Union Directive (2010/63/EU), and institutional ethical guidelines (E-67-218-38). The animals were maintained in the Chronobiotron animal facility (UMS 3415, Strasbourg) under a 12-hour/12-hour light dark (LD) cycle (300 lux [lx] and <5 lx dim red light during light and dark, respectively) at a 22 ± 1°C ambient temperature, with free access to food and water. Mice carrying the PER2::LUC reporter^6^ were bred either on a pigmented C57BL/6J (Charles River Laboratories, France) or albino RjOrl:SWISS (Janvier Labs, Le Genest-Saint-Isle, France) background. We used both male and female mice. We used age-matched 7 to 11-week-old PER2::LUC mice maintained on C57BL/6J background. We also included 7 to 22-week-old age-matched mice in experiments involving both C57BL/6J and RjOrl:SWISS backgrounds (Fig. 4). In Figures 1, 2, and 4, we used explants obtained from homozygous *mPer2^Luc^* mice, whereas, in Figure 5, we obtained explants from heterozygous *mPer2^Luc^*^/+^ mice due to difficulties in breeding animals. In each experiment, the mice were euthanized at the same time of day, but the exact sampling times differed between experiments. The euthanization times ranged between ZT 3.75 and 10 but, for samples that were compared within a figure, the difference in sampling time did not exceed 3 hours. This interval was notably due to the time needed to collect and process all eyes. Euthanasia was performed by cervical dislocation, the eyes were enucleated and placed in HBSS media containing 4.2 mM sodium bicarbonate (Sigma S8761), 10 mM N-2-hydroxyethylpiperazine-N'-2-ethanesulfonic acid (Sigma H0887), 100 U/mL of penicillin, and 100 µg/mL of streptomycin (Sigma P4333), as described previously.^17^^,^^22^^,^^23^

### Sample Preparation

Dissections were performed in HBSS media at room temperature. A sterile needle was used to puncture the eyeball under the ora serrata, and surgical scissors were used to cut around it. The lens and iris were dissected out of the cornea. The retina was excised from the PEC. The PEC was prepared by removing the muscle tissue and optic nerve lying on the basal side. Removal of the RPE from the PEC was performed by scraping of PECs by a curved blade and forceps. The retina, PEC, and cornea were radially incised, flattened, and placed on a semipermeable membrane (PICMORG50; EMD Millipore, Billerica, MA, USA) in a 35 mm culture dish.

### Bioluminescence Recordings

Retinal samples were preincubated for 24 hours at 37°C in a humidified 5% CO_2_ atmosphere in 1 mL of Neurobasal A medium (Invitrogen, Life Technologies, Carlsbad, CA, USA) containing antibiotics (25 U/mL penicillin and 25 mg/mL streptomycin), 2% B27 (Invitrogen, Life Technologies), and 2 mM L-glutamine. Then, the medium was replaced by 1 mL of medium 199 (Sigma-Aldrich, St. Louis, MO, USA) containing antibiotics (25 U/mL penicillin and 25 mg/mL streptomycin), 4 mM sodium bicarbonate, 20 mM D (+)-glucose, 2% B27, 0.7 mM L-glutamine, and 100 µM beetle luciferin (Promega, Fitchburg, WI, USA), and the dishes were sealed (Dow Corning high-vacuum grease; Midland, MI, USA) under normal air and placed into the LumiCycle (Actimetrix, Wilmette, IL, USA) heated at 36°C. The procedure was modified for the mouse PECs and corneas: preincubation for 24 hours and medium change was done in full 199 medium. All medium changes were performed under dim red light. Samples were recorded during at least 5 days and the photons were counted during 112 seconds every 15 minutes.

Oscillations were analyzed using the Lumicycle Analysis software (Actimetrics, USA). Raw data (counts per second [cps]) were baseline-subtracted (24hours running average, based on the whole recording period). LMFit (damped sine) function was used to calculate the periods and amplitudes with the following criteria for selecting the intervals: a minimum of at least 3 cycles in length (except for a few female retina samples in Figures 1 and 2 where 2 cycles were used), up to 5 cycles, the first oscillation was excluded from the analysis and the selected interval had the maximum goodness of fit. The time of the first peak in the recordings is considered the acrophase and was expressed by considering that ZT0 = lights on and ZT12 = lights off. The relative rhythmic power was determined using the Periodogram function of the Lumicycle Analysis to evaluate the robustness of the rhythms.^22^^,^^24^^–^^27^

### Cryosection, Immunolabeling, and Microscopy

The eyes were obtained from C57BL/6J mice, embedded in OCT (Tissue-Tek O.C.T. Compound, USA) and cryo-sectioned. Cryostat sections (10 µm) were permeabilized with Triton (PBS-BSA 1% and Triton X-100 0.4%, for 5 minutes) and blocked with saturation buffer (PBS-BSA 1% and 0.03% Triton X-100 in PBS) for 60 minutes. The sections were incubated overnight with primary anti-BMAL1 (NB100-2288, Novus; 1/500) diluted in saturation buffer, washed 2 × 5 minutes in PBS, incubated with goat anti-rabbit IgG- Alexa 488 secondary antibodies (2 hours at room temperature) and washed again for 40 minutes. The cell nuclei were stained with DAPI (Molecular Probes). The slides were washed 3 × 5 minutes in PBS, mounted in PBS/glycerol (1:1), and scanned in a NanoZoomer (Hamamatsu Photonics) at 40× and viewed using NDP.view2 software (Hamamatsu Photonics).

### Statistics

Individual data points are plotted together with the means ± SD. Plots were generated using GraphPad Prism software (version 8.3.0; La Jolla, CA, USA). Normality of distribution was confirmed using the Kolmogorov-Smirnov test. One-way ANOVA analysis was performed in Figure 2. Two-way ANOVA analyses were performed in this study with the following factors: Figure 1 = sex and tissue type; Figure 4 = mouse background and tissue type; and Figure 5 = preincubation medium and tissue type. Further analyses, where indicated, were performed using the Holm-Sidak's post hoc tests. Student's *t*-test was performed in Supplementary Figure S1.

## Results

### Sex Does not Affect Circadian Rhythms of PER2::LUC Bioluminescence of Ex Vivo Ocular Tissues

**Figure 3.**
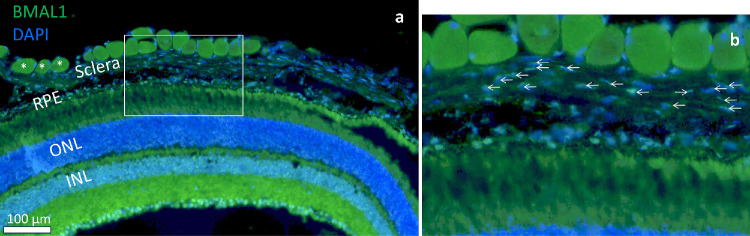
**Immunohistochemistry of murine retina.** Representative micrograph of BMAL1 and DAPI labeling in the murine (**a**) retina. Extraocular muscle fibers that show strong background labeling, are indicated by *asterisks*. (**b**) Zoomed-in view of BMAL1 and DAPI labeling in the sclera. *Arrows* indicate BMAL1 positive nuclei. The scale bar is 100 µm. INL, inner nuclear layer; ONL, outer nuclear layer; RPE, retinal pigment epithelium.

We studied the effect of sex on ocular clocks in mice. We recorded PER2::LUC bioluminescence from C57BL/6J male mice retinas (see Fig. 1a), PECs (see Fig. 1b), and corneas (see Fig. 1c), and female mice retinas (see Figs. 1d–f). To maintain retinal viability throughout the recording sessions, retinal explants were preincubated with Neurobasal A medium for 24 hours, followed by a medium change using M199 medium.^9^ Conversely, PECs and corneas were preincubated with M199 medium for 24 hours and had a medium change with the same medium as recommended by Baba and Tosini.^7^ The same culturing was performed in further experiments (see Supplementary Fig. S1; Figs. 2, 3). Two-way ANOVA testing revealed that sex was not a statistically significant factor for period length (see Fig. 1g; F(1, 44) = 0.013, *P* = 0.91), amplitude (see Fig. 1h; F(1, 44) = 3.48, *P* = 0.069), acrophase (see Fig. 1i; F(1, 44) = 2.78, *P* = 0.10), and relative rhythmic power (see Fig. 1j; F(1, 45) = 1.03, *P* = 0.32) of PER2::LUC bioluminescence rhythms in C57BL/6J mice. We also studied PER2::LUC bioluminescence rhythms of male mice (see Supplementary Fig. S1a) and female mice (Supplementary Fig. S1b) RjOrl:SWISS PECs. Similarly, there were no statistically significant differences in period length (see Supplementary Fig. S1c; Student's *t*-test, t(15) = 0.23, *P* = 0.82), amplitude (see Supplementary Fig. S1d; Student's *t*-test, t(15) = 0.22, *P* = 0.83), acrophase (see Supplementary Fig. S1e; Student's *t*-test, t(15) = 1.88, *P* = 0.08), and relative rhythmic power (see Supplementary Fig. S1f; Student's *t*-test, t(15) = 0.47, *P* = 0.65) between male and female PECs of RjOrl:SWISS mice. Overall, these results suggest that sex does not influence ocular clocks in mice. Bearing this conclusion in mind, we included results obtained from samples of both sexes in further experiments.

### Circadian Rhythms of PER2::LUC Bioluminescence in Ex Vivo Ocular Tissues: A Clock in the Sclera?

We initially recorded PER2::LUC bioluminescence from corneas (see Fig. 2a), PECs (see Fig. 2b), and retinas (see Fig. 2d) obtained from PER2::LUC mice on the C57BL/6J background. Previously published work has implied that the RPE is the main driver of bioluminescence rhythms in PECs.^12^ Thus, we included PECs with the RPE removed (scraped off) as negative controls. Unexpectedly, we observed that sclerae showed circadian oscillations of PER2::LUC bioluminescence (see Fig. 2c). We compared the rhythm in the sclera to oscillators of other ocular tissues: the cornea, the PEC, and the retina (see Figs. 2e–h). To increase statistical power, we pooled data from 7 experiments with ocular tissues being obtained from a total of 25 mice. One-way ANOVA analyses revealed that period length (see Fig. 2e; F(3, 42) = 20.75, *P* < 0.0001), amplitude (see Fig. 2f; F(3, 42) = 40.02, *P* < 0.0001), acrophase (see Fig. 2g; F(3, 42) = 19.53, *P* < 0.0001), and relative rhythmic power (see Fig. 2h; F(3, 43) = 3.14, *P* = 0.035) differed significantly between bioluminescence rhythms of different ocular tissues. Holm-Sidak's post hoc tests showed that scraped PECs had significantly longer periods compared with corneas (*P* < 0.0001), intact PECs (*P* < 0.0001), and retinas (*P* < 0.0001). In addition, the bioluminescence rhythms of scraped PECs had significantly lower amplitudes compared with the ones of retinas (*P* < 0.0001) and tended to be lower than for the corneas (*P* = 0.051). The acrophases of scraped PECs rhythms differed significantly compared with the corneas (*P* < 0.0001), intact PECs (*P* < 0.0001), and retinas (*P* < 0.0001). We performed immunohistochemical analysis using C57BL6/J mice to provide further evidence that the sclera harbors a circadian oscillator (Fig. 3a). In the sclera, we found labeling positive for the protein product of the core clock gene, BMAL1 (Fig. 3b). These results suggest that the sclera contains a circadian oscillator with lower amplitudes, longer periods, and distinct acrophases compared with other ocular tissues.

### Does Mouse Strain Affect Circadian Rhythms of PER2::LUC Bioluminescence of Ex Vivo Ocular Tissues?

**Figure 4.**
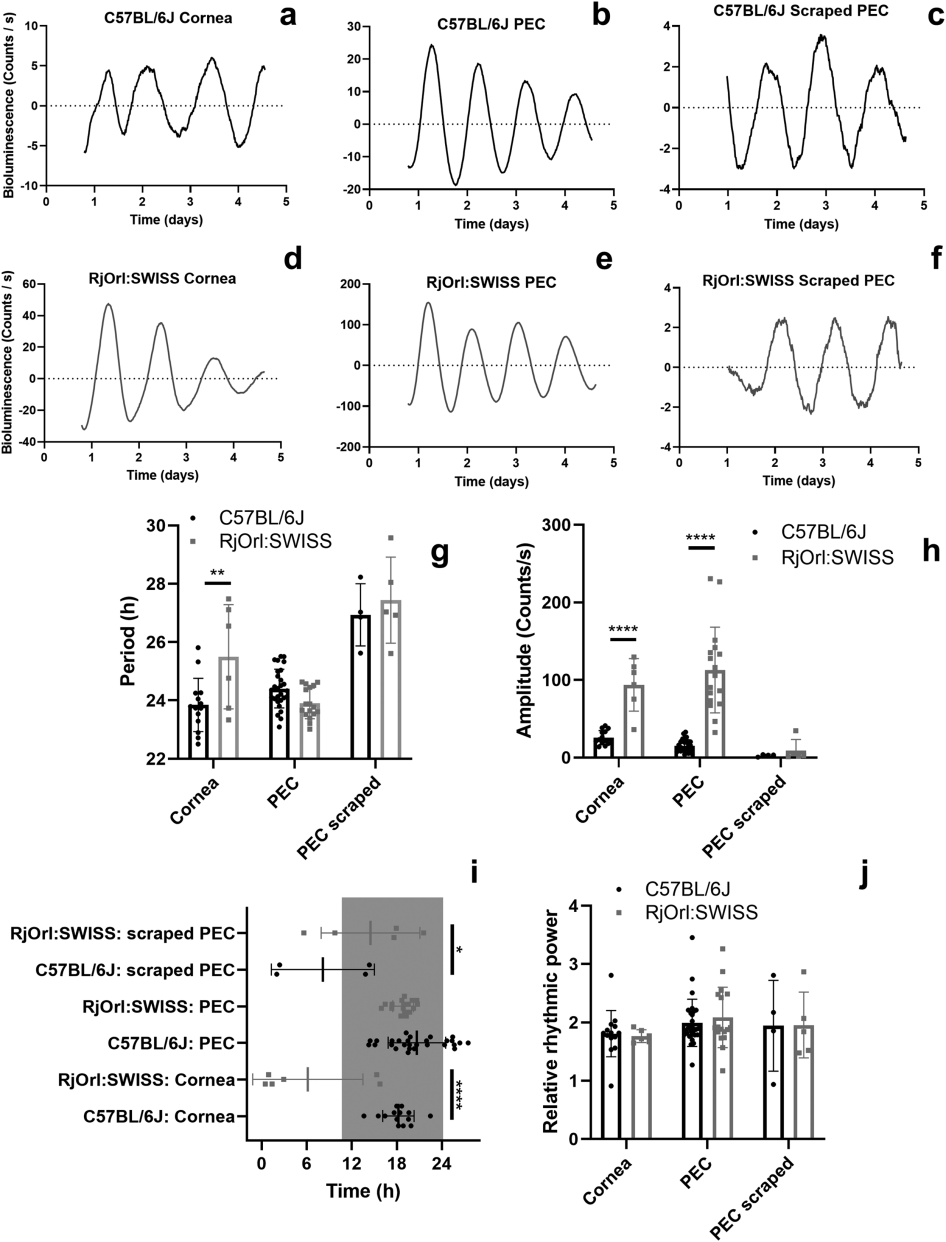
**Mouse background influences circadian rhythms in ocular tissues**. Representative examples of bioluminescence rhythms in the corneas, intact PECs, and scraped PECs obtained from PER2::LUC mice maintained on a (**a**–**c**) C57BL/6J background and, respectively, from a (**d**–**f**) RjOrl:SWISS background. Two-way ANOVA analysis was used to study the effects of background on (**g**) period length, (**h**) amplitude, (**i**) time of first peak, that is, acrophase, and (**j**) relative rhythmic power of PER2::LUC bioluminescence oscillations in the corneas, and intact and scraped PECs. Time in (**i**) is projected ZT with ZT12 = lights off. The *gray rectangle* represents subjective nighttime. Individual data points are plotted together with means ± SD. Holm-Sidak's post hoc test comparison, *P < 0.05, **P < 0.01, ****P < 0.0001. PEC, posterior eye cup. *N* = 3 to 28.

We speculated that mouse background may influence ocular rhythms. To test this hypothesis, we measured bioluminescence rhythms from the corneas (see Figs. 4a, 4d), PECs (see Figs. 4b, 4e), and scraped PECs (see Figs. 4c, 4f) from PER2::LUC mice maintained on two backgrounds: the pigmented one, C57BL/6J, and the albino line RjOrl:SWISS. Two-way ANOVA testing revealed that the mouse background was a significant factor for period length (see Fig. 4g; F(1, 68) = 4.29, *P* = 0.0421), amplitude (see Fig. 4h; F(1, 68) = 43.95, *P* < 0.0001), and acrophase (see Fig. 4i; F(1, 68) = 4.64, *P* = 0.035), but not relative rhythmic power (see Fig. 4j; F(1, 68) = 0.023, *P* = 0.88) of PER2::LUC bioluminescence rhythms. In particular, Holm-Sidak's post hoc testing revealed that corneas of RjOrl:SWISS mice have significantly longer periods compared with the corneas of C57BL/6J mice (see Fig. 4g, *P* = 0.011). Furthermore, PECs (see Fig. 4h; *P* < 0.0001) and corneas (see Fig. 4h; *P* < 0.0001) of RjOrl:SWISS mice have significantly higher amplitudes compared with the ones of C57BL/6J mice. Holm-Sidak's post hoc testing revealed that corneas (see Fig. 4i; *P* < 0.0001) and scraped PECs (see Fig. 4i; *P* = 0.039) of RjOrl:SWISS mice have significantly different acrophases compared with the ones of C57BL/6J mice. These results show that the mouse background significantly affects PER2::LUC bioluminescence rhythms of ocular tissues. Among the studied ocular tissues, the rhythms in corneas were most affected by the mouse background.

### Neurobasal A Medium Preincubation Enhances Circadian Rhythms of PER2::LUC Bioluminescence of Ex Vivo Ocular Tissues

**Figure 5.**
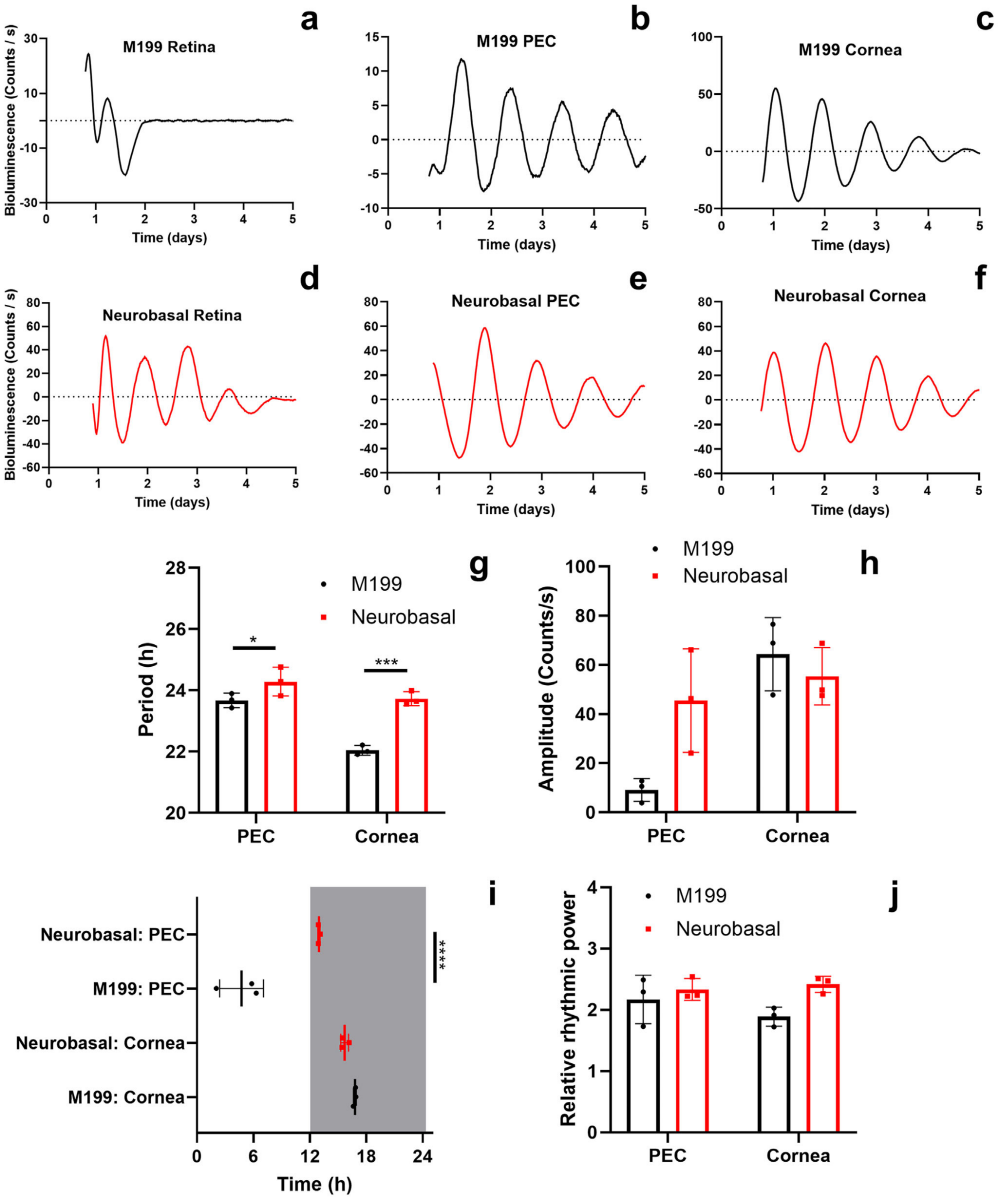
**Preincubation medium affects circadian rhythms of PER2::LUC bioluminescence of ex vivo ocular tissues.** Ocular tissues were obtained from heterozygous C57BL/6J *mPer2^Luc^*^/+^ mice. Representative examples of PER2::LUC bioluminescence rhythms in the retinas, PECs, and corneas that were preincubated for 24 hours with (**a**–**c**) M199 and (**d**–**f**) Neurobasal A medium. (**g**) Period length, (**i**) acrophase, and (**j**) relative rhythmic power in ocular tissues was affected by the choice of preincubation medium. Conversely, (**h**) amplitude of PER2::LUC bioluminescence rhythms in ocular tissues was not affected by the choice of preincubation medium. Time in (**i**) is projected ZT with ZT12 = lights off. The *gray rectangle* represents subjective nighttime. Individual data points are plotted together with means ± SD. Holm-Sidak's post hoc test comparison, *P < 0.05, ****P < 0.0001. PEC, posterior eye cup. *N* = 3.

A 24-hour preincubation with Neurobasal A medium is used for maintaining retinal explant viability for PER2::LUC bioluminescence recordings.^9^ Such a procedure is not necessary for studying rhythms in PECs^12^^,^^15^^,^^23^^,^^28^ and corneas.^11^^,^^28^ We tested the hypothesis that Neurobasal A medium preincubation influences PER2::LUC bioluminescence rhythms in ocular tissues. Retinas, PECs, and corneas were obtained from heterozygous C57BL/6J *mPer2^Luc^*^/+^ mice. These tissues were preincubated for 24 hours with either recording medium, M199 (representative PER2::LUC bioluminescence recordings of the retina, the PECs, and the cornea: see Figs. 5a–c) or Neurobasal A (the retina, the PECs, and the cornea: see Figs. 5d–f), followed by a medium change in all samples with recording medium containing M199. At least 3 complete cycles were observed in all bioluminescence recordings, except in the ones recorded from M199 preincubated retinas whose oscillations were highly variable and attenuated rapidly (see Fig. 5a). Due to this reason, we could not accurately measure parameters of oscillations in M199 preincubated retinas. Thus, it was not possible to statistically compare Neurobasal A and M199 preincubated retinas. Conversely, such comparisons were possible for PEC and cornea recordings. Two-way ANOVA testing revealed that the choice of preincubation medium was a significant factor for period length (see Fig. 5g; F(1, 8) = 44.82, *P* = 0.0002), acrophase (see Fig. 5i; F(1, 8) = 27.01, *P* = 0.0008), and relative rhythmic power of PER2::LUC bioluminescence rhythms (see Fig. 5j; F(1, 8) = 6.19, *P* = 0.038), but not amplitude (see Fig. 5h; F(1, 8) = 2.72, *P* = 0.14). Holm-Sidak's post hoc testing revealed that PECs preincubated with Neurobasal A have significantly longer periods (*P* = 0.036) and distinct acrophases (*P* < 0.0001) compared with PECs preincubated with M199 medium. Although the 2-way ANOVA analysis did not show an effect of preincubation medium on amplitudes, Holm-Sidak's post hoc test showed higher amplitudes (*P* = 0.029) of Neurobasal A preincubated PECs compared with M199 treated ones. We also found that corneas preincubated with Neurobasal A medium have significantly longer periods compared with the ones preincubated with M199 medium (*P* = 0.0002). These results show that Neurobasal A medium can enhance circadian rhythmicity not only in the retinas, but also in other ocular tissues, such as the PECs and corneas.

## Discussion

In this study, we characterized PER2::LUC bioluminescence rhythms in various ocular tissues: corneas, retinas, and PECs, and discovered a hitherto unknown oscillator in the sclera. We did not observe any significant effect of sex in PER2::LUC expression in ocular tissues. Further, we showed the significance that *mPer2^Luc^* mouse background plays in bioluminescence rhythms of ocular clocks. Finally, we found that culturing conditions, such as Neurobasal A medium preincubation, can substantially influence rhythms of PER2::LUC bioluminescence of most ocular tissues.

Several lines of evidence suggested that ocular rhythms may differ between sexes. The diurnal variation of intraocular pressure and tear production was shown to vary between sexes.^29^^,^^30^ Androgen, estrogen, and the progesterone receptor mRNA were found in a variety of ocular tissues.^31^^,^^32^ In certain tissue types, such as livers and adrenal glands, there were sex differences in PER2::LUC rhythms.^33^ However, we did not find any significant sex differences in PER2::LUC bioluminescence rhythms of retinal, corneal, and PEC explants. Calligaro and colleagues also showed no effect of sex on retinal rhythms.^21^ Although our results show that, between sexes, there are no intrinsic differences in ocular rhythms, we cannot exclude the possibility that sex hormones may modulate these rhythms.

Prior work on the regulation of the murine RPE clock was conducted using bioluminescence recordings of PEC explants from *Per2^Luc^* mice.^12^^,^^15^^,^^23^^,^^28^ It was implied that scraping the RPE cell layer from the PEC attenuates oscillations in the explant, indicating that the RPE is the source of rhythms of PER2::LUC bioluminescence in the PEC.^12^ Unexpectedly, we found that scraped PECs *did* show oscillations of PER2::LUC bioluminescence, suggesting that the sclera also contains a clock. These oscillations had lower amplitudes, longer periods, and distinct acrophases compared with other ocular tissues, suggesting that this is a weak and less coupled oscillator^34^ compared with other ocular clocks. The source of these rhythms is not entirely clear. The low amplitudes could be due to a small signal from many cells or a high amplitude rhythm that originates from structures that are low in number. We can speculate about three possibilities regarding the source of bioluminescence rhythms: (1) an oscillator within the sclera, (2) noradrenergic^35^ and/or cholinergic^36^ autonomic axons that innervate the ciliary body and iris, and (3) extraocular muscle fibers. Our data supports possibility one, namely, the immunolabeling experiment confirms the presence of the core clock component BMAL1 in the sclera. The sclera is comprised of collagen-rich scleral extracellular matrix (ECM) and sparsely populated resident cells, called fibrocytes.^37^ Unfortunately, there is a lack of specific markers for fibrocytes and their localization is sparse.^38^ However, it is known that, upon insult, fibrocytes can undergo transformation into active fibroblasts,^37^ a cell type known to show robust circadian oscillations.^39^ Therefore, it is likely that scleral rhythms are generated by resident fibrocytes. Possibility two is unlikely because neurons require a 24-hour Neurobasal A preincubation to sustain PER2::LUC bioluminescence rhythms^9^ and the recordings of scraped PECs were performed without Neurobasal A. Possibility three is unlikely because we meticulously removed muscle tissue from PEC explants prior to recordings. Regardless of its source, we do not know the role the scleral clock plays in overall eye physiology. In the cartilaginous avian sclera, chondrocytes show a circadian rhythm in proteoglycan synthesis, which is suggested to drive the rhythm in ocular elongation.^40^ In the mammalian sclera, it remains unclear whether fibrocytes and/or fibroblasts show rhythms of ECM component synthesis which might underly diurnal variations of ocular dimensions.^41^^–^^44^

We found that bioluminescence of the corneas, retinas, and PECs oscillated with a periodicity of roughly 24 hours, which is consistent with previous work.^28^ In addition, the acrophase of retinal rhythms occurred roughly in the middle of the night, as reported by others.^16^^,^^28^ Conversely, the peak timing of PEC bioluminescence rhythms is slightly shifted in comparison with the literature,^28^ whereas the acrophase of corneal rhythms varies depending on the study.^16^^,^^28^ The reason for such variability might be due to high dispersion of our data and/or the methods for calculating the acrophase. However, we think it is most likely due to differences in culturing protocols. In support of this explanation, we found that Neurobasal A medium preincubation can substantially influence PER2::LUC bioluminescence rhythms in ocular tissues. This protocol is widely used to enable long-term culture of retinal explants for monitoring PER2::LUC expression,^9^^,^^18^^,^^21^^,^^27^^,^^45^ which was also essential for our prior work.^17^^,^^18^^,^^22^^,^^24^ Our results showed that Neurobasal A medium preincubated retinas exhibited consistent and robust oscillations. Neurobasal A medium preincubation increased the period lengths and altered the acrophases of corneal and PEC rhythms compared to the ones preincubated with M199. However, it was previously reported that recording PER2::LUC bioluminescence rhythms in retinal explants was possible with only M199 medium.^7^^,^^10^^,^^28^ We found that bioluminescence rhythms in retinas preincubated with M199 medium gave highly variable rhythms which quickly attenuated. Conversely, in Figures 1, 2, and 4, the PEC and cornea explants still showed reasonably stable and consistent oscillations despite being cultured in M199 medium, as recommended by Baba and colleagues.^7^^,^^11^^,^^12^ These effects could be explained by the fact that Neurobasal A medium contains a concentration of components optimized for culturing neurons.^46^

Most work using *Per2^Luc^* mice to study ocular clocks was done on a C57BL6 background.^9^^–^^12^^,^^15^^,^^17^^,^^19^^,^^20^^,^^22^^–^^24^^,^^27^^,^^28^^,^^47^ There is a limited number of studies in which a different mouse strain was used to study ocular clocks.^9^^,^^16^ These studies have used such backgrounds as a strategy to overcome the main limitation of the C57BL6 in chronobiology, that is, the inability of these mice to secrete the clock regulatory hormone melatonin.^48^ Recent work suggests that the effect of background on ocular clock function can be unrelated to melatonin deficiency. Specifically, C57BL/6 and 129T2/SvEmsJ mice are both deficient in melatonin,^48^^–^^50^ and they showed differences in timing of phagocytosis of photoreceptor outer segments,^51^^–^^53^ a known circadian clock-regulated process in the eye.^54^ Differences in pigmentation are a strain-related feature that affects circadian rhythms in mice that are unrelated to the strain's melatonin status.^55^ In this paper, we studied ocular clocks using the albino RjOrl:SWISS background,^56^ a line which has a similar free-running period as C57BL/6.^57^ Our results showed significant differences in bioluminescence rhythms in ocular tissues of albino RjOrl:SWISS compared with pigmented C57BL/6J *mPer2^Luc^* mice. RjOrl:SWISS corneas and PECs had higher amplitudes of PER2::LUC bioluminescence compared with the ones of C57BL/6J mice. Corneal bioluminescence of RjOrl:SWISS oscillated with a different phase and longer period compared with the ones of C57BL/6J mice suggesting that corneal clocks are more affected by pigment mutation. These differences may be due to technical or biological reasons. It is possible that the pigment of C57BL/6J ocular tissues may confound the PER2 bioluminescence signal, leading to lower amplitudes in these explants, as observed in our results. This hypothesis does not explain the differences in corneal rhythms because the cornea is transparent.^58^ To the best of our knowledge, the ability of RjOrl:SWISS to synthesize melatonin is not known. Melatonin secretion may explain the differences in corneal clocks between the strains, as the corneal clock is set by this hormone.^11^^,^^16^

There are limitations in our study. Our strategy for calculating acrophases is likely affected by period length. We also observed high variability in estimating the acrophase in scleral samples (see Figs. 2g, 4i). Despite our best efforts, we cannot fully exclude the possibility that these samples may contain remnants of RPE or choroid. The outliers have similar acrophases as intact PECs, which supports this possibility. By contrast, period lengths remained consistent in scleral rhythms, suggesting that they originate from the same cell type. We also cannot exclude the possibility that the scraping procedure could have affected PER2::LUC bioluminescence rhythms. A further limitation is the relatively wide age range of mice used in this study (7–22 weeks). It is reported that there are limited differences in PER2::LUC bioluminescence rhythms of retinas of mice aged 2 to 3 months compared with the ones aged up to 8 months.^21^ Thus, it is unlikely that age differences confounded our results because we used age-matched mice with the youngest ones being approximately 2 months old. Next, we used homozygous *mPer2^Luc^* mice for experiments shown in Figures 1, 2, and 4, whereas heterozygous *mPer2^Luc^*^/+^ mice in Figure 5 were due to difficulties in breading homozygous mice. It is known that the *Luc* modification in *mPer2^Luc^* mice results in broad changes in the regulation and overt expression of circadian rhythmicity, with homozygous *mPer2^Luc/Luc^* exhibiting lengthened free-running rhythms under constant darkness (DD) relative to wild type (WT) littermates and *mPer2^Luc/+^* showing an intermediate phenotype with period instability in DD.^59^ Thus, these allele differences may explain the observed discrepancies in acrophases of ocular tissues in Figures 2 and 4 compared with Figure 5, slightly earlier periods in Figure 5 compared to Figures 2 and 4; discrepancies in amplitude in Figure 5 compared with Figures 1, 2, and 4. However, we used the experiments in Figure 5 to study the effects of culturing conditions on PER2::LUC bioluminescence. Thus, the observed discrepancies did not affect our conclusions.

This work opens the possibility for various future directions. The identification of cell types generating scleral rhythms, the regulation of this oscillator, and the physiological functions, which are under its control, remain open questions. The component in Neurobasal A medium that modulates ocular rhythms remains elusive. We do not know the molecular mechanisms underlying the differences in ocular rhythms between mouse strains. These, along with other questions, remain topics for future investigation.

## Supplementary Material

Supplement 1

## References

[1] Cox KH, Takahashi JS. Circadian clock genes and the transcriptional architecture of the clock mechanism. *J Mol Endocrinol*. 2019; 63: R93–R102. 31557726 10.1530/JME-19-0153 PMC6872945

[2] Cox KH, Takahashi JS. Introduction to the Clock System. *Adv Exp Med Biol*. 2021; 1344: 3–20. 34773223 10.1007/978-3-030-81147-1_1

[3] Takahashi JS. Molecular components of the circadian clock in mammals. *Diabetes Obes Metab*. 2015; 17(Suppl 1): 6–11. 26332962 10.1111/dom.12514 PMC4560116

[4] McMahon DG, Iuvone PM, Tosini G. Circadian organization of the mammalian retina: from gene regulation to physiology and diseases. *Prog Retin Eye Res*. 2014; 39: 58–76. 24333669 10.1016/j.preteyeres.2013.12.001 PMC3927986

[5] Felder-Schmittbuhl MP, Buhr ED, Dkhissi-Benyahya O, et al. Ocular clocks: adapting mechanisms for eye functions and health. *Invest Ophthalmol Vis Sci*. 2018; 59: 4856–4870. 30347082 10.1167/iovs.18-24957 PMC6181243

[6] Yoo SH, Yamazaki S, Lowrey PL, et al. PERIOD2::LUCIFERASE real-time reporting of circadian dynamics reveals persistent circadian oscillations in mouse peripheral tissues. *Proc Natl Acad Sci USA*. 2004; 101: 5339–5346. 14963227 10.1073/pnas.0308709101 PMC397382

[7] Baba K, Tosini G. Real-time monitoring of circadian rhythms in the eye. *Methods Mol Biol*. 2022; 2550: 367–375. 36180706 10.1007/978-1-0716-2593-4_37

[8] Tosini G, Menaker M. Circadian rhythms in cultured mammalian retina. *Science (New York, NY)*. 1996; 272: 419–421. 10.1126/science.272.5260.419 8602533

[9] Ruan GX, Allen GC, Yamazaki S, McMahon DG. An autonomous circadian clock in the inner mouse retina regulated by dopamine and GABA. *PLoS Biol*. 2008; 6: e249. 18959477 10.1371/journal.pbio.0060249 PMC2567003

[10] Ruan GX, Zhang DQ, Zhou T, Yamazaki S, McMahon DG. Circadian organization of the mammalian retina. *Proc Natl Acad Sci USA*. 2006; 103: 9703–9708. 16766660 10.1073/pnas.0601940103 PMC1480470

[11] Baba K, Davidson AJ, Tosini G. Melatonin entrains PER2::LUC bioluminescence circadian rhythm in the mouse cornea. *Invest Ophthalmol Vis Sci*. 2015; 56: 4753–4758. 26207312 10.1167/iovs.15-17124 PMC4516012

[12] Baba K, Sengupta A, Tosini M, Contreras-Alcantara S, Tosini G. Circadian regulation of the PERIOD 2::LUCIFERASE bioluminescence rhythm in the mouse retinal pigment epithelium-choroid. *Mol Vis*. 2010; 16: 2605–2611. 21151601 PMC3000237

[13] Besharse JC, McMahon DG. The retina and other light-sensitive ocular clocks. *J Biol Rhythms*. 2016; 31: 223–243. 27095816 10.1177/0748730416642657 PMC5479307

[14] Tsuchiya S, Buhr ED, Higashide T, Sugiyama K, Van Gelder RN. Light entrainment of the murine intraocular pressure circadian rhythm utilizes non-local mechanisms. *PLoS One*. 2017; 12: e0184790. 28934261 10.1371/journal.pone.0184790 PMC5608236

[15] Baba K, DeBruyne JP, Tosini G. Dopamine 2 receptor activation entrains circadian clocks in mouse retinal pigment epithelium. *Sci Rep*. 2017; 7: 5103. 28698578 10.1038/s41598-017-05394-x PMC5505969

[16] Huynh AV, Buhr ED. Melatonin adjusts the phase of mouse circadian clocks in the cornea both ex vivo and in vivo. *J Biol Rhythms*. 2021; 36: 470–482. 34323103 10.1177/07487304211032385 PMC8811878

[17] Jaeger C, Sandu C, Malan A, Mellac K, Hicks D, Felder-Schmittbuhl MP. Circadian organization of the rodent retina involves strongly coupled, layer-specific oscillators. *FASEB J*. 2015; 29: 1493–1504. 25573753 10.1096/fj.14-261214

[18] Calligaro H, Coutanson C, Najjar RP, et al. Rods contribute to the light-induced phase shift of the retinal clock in mammals. *PLoS Biol*. 2019; 17: e2006211. 30822304 10.1371/journal.pbio.2006211 PMC6415865

[19] Buhr ED, Yue WW, Ren X, et al. Neuropsin (OPN5)-mediated photoentrainment of local circadian oscillators in mammalian retina and cornea. *Proc Natl Acad Sci USA*. 2015; 112: 13093–13098. 26392540 10.1073/pnas.1516259112 PMC4620855

[20] Díaz NM, Lang RA, Van Gelder RN, Buhr ED. Wounding induces facultative Opn5-dependent circadian photoreception in the murine cornea. *Invest Ophthalmol Vis Sci*. 2020; 61: 37. 10.1167/iovs.61.6.37 PMC7415322 32543667

[21] Calligaro H, Kinane C, Bennis M, Coutanson C, Dkhissi-Benyahya O. A standardized method to assess the endogenous activity and the light-response of the retinal clock in mammals. *Mol Vis*. 2020; 26: 106–116. 32180677 PMC7058435

[22] Gegnaw ST, Sandu C, Mendoza J, Bergen AA, Felder-Schmittbuhl MP. Dark-adapted light response in mice is regulated by a circadian clock located in rod photoreceptors. *Exp Eye Res*. 2021; 213: 108807. 34695438 10.1016/j.exer.2021.108807

[23] Milicevic N, Mazzaro N, de Bruin I, et al. Rev-erbalpha and photoreceptor outer segments modulate the circadian clock in retinal pigment epithelial cells. *Sci Rep*. 2019; 9: 11790. 31409842 10.1038/s41598-019-48203-3 PMC6692399

[24] Gegnaw ST, Sandu C, Mazzaro N, Mendoza J, Bergen AA, Felder-Schmittbuhl MP. Enhanced robustness of the mouse retinal circadian clock upon inherited retina degeneration. *J Biol Rhythms*. 2022; 37: 567–574. 35912966 10.1177/07487304221112845

[25] Buonfiglio DC, Malan A, Sandu C, et al. Rat retina shows robust circadian expression of clock and clock output genes in explant culture. *Mol Vis*. 2014; 20: 742–752. 24940028 PMC4043612

[26] Herzog ED, Kiss IZ, Mazuski C. Measuring synchrony in the mammalian central circadian circuit. *Methods Enzymol*. 2015; 552: 3–22. 25707270 10.1016/bs.mie.2014.10.042 PMC5110928

[27] Ruan GX, Gamble KL, Risner ML, Young LA, McMahon DG. Divergent roles of clock genes in retinal and suprachiasmatic nucleus circadian oscillators. *PLoS One*. 2012; 7: e38985. 22701739 10.1371/journal.pone.0038985 PMC3372489

[28] Baba K, Tosini G. Aging alters circadian rhythms in the mouse eye. *J Biol Rhythms*. 2018; 33: 441–445. 29940798 10.1177/0748730418783648 PMC6398161

[29] Kulualp K, Kilic S, Aytekin O. Effects of sex, eye-side, diurnal variation on intraocular pressure in calves. *Pol J Vet Sci*. 2019; 22: 67–74. 30997760 10.24425/pjvs.2018.125609

[30] Piccione G, Giannetto C, Fazio F, Giudice E. Daily rhythm of tear production in normal horse. *Vet Ophthalmol*. 2008; 11(Suppl 1): 57–60. 19046271 10.1111/j.1463-5224.2008.00647.x

[31] Wickham LA, Gao J, Toda I, Rocha EM, Ono M, Sullivan DA. Identification of androgen, estrogen and progesterone receptor mRNAs in the eye. *Acta Ophthalmol Scand*. 2000; 78: 146–153. 10794246 10.1034/j.1600-0420.2000.078002146.x

[32] Kobayashi K, Kobayashi H, Ueda M, Honda Y. Estrogen receptor expression in bovine and rat retinas. *Invest Ophthalmol Vis Sci*. 1998; 39: 2105–2110. 9761289

[33] Kuljis DA, Loh DH, Truong D, et al. Gonadal- and sex-chromosome-dependent sex differences in the circadian system. *Endocrinology*. 2013; 154: 1501–1512. 23439698 10.1210/en.2012-1921 PMC3602630

[34] Schmal C, Herzog ED, Herzel H. Measuring relative coupling strength in circadian systems. *J Biol Rhythms*. 2018; 33: 84–98. 29219034 10.1177/0748730417740467 PMC6344889

[35] Tervo T. Demonstration of adrenergic nerve fibres in the nasociliary but not the ophthalmic nerve of the rat. *Exp Eye Res*. 1978; 27: 607–613. 720434 10.1016/0014-4835(78)90145-8

[36] Mahran ZY, Sakla FB. The pattern of innervation of the extrinsic ocular muscles and the intra-orbital ganglia of the albino mouse. *Anat Rec*. 1965; 152: 173–183. 4221069 10.1002/ar.1091520208

[37] Boote C, Sigal IA, Grytz R, Hua Y, Nguyen TD, Girard MJA. Scleral structure and biomechanics. *Prog Retin Eye Res*. 2020; 74: 100773. 31412277 10.1016/j.preteyeres.2019.100773 PMC7187923

[38] Reinhardt JW, Breuer CK. Fibrocytes: a critical review and practical guide. *Front Immunol*. 2021; 12: 784401. 34975874 10.3389/fimmu.2021.784401 PMC8718395

[39] Balsalobre A, Damiola F, Schibler U. A serum shock induces circadian gene expression in mammalian tissue culture cells. *Cell*. 1998; 93: 929–937. 9635423 10.1016/s0092-8674(00)81199-x

[40] Nickla DL, Rada JA, Wallman J. Isolated chick sclera shows a circadian rhythm in proteoglycan synthesis perhaps associated with the rhythm in ocular elongation. *J Comp Physiol A*. 1999; 185: 81–90. 10450612 10.1007/s003590050368

[41] Nickla DL. Ocular diurnal rhythms and eye growth regulation: where we are 50 years after Lauber. *Exp Eye Res*. 2013; 114: 25–34. 23298452 10.1016/j.exer.2012.12.013 PMC3742730

[42] Burfield HJ, Patel NB, Ostrin LA. Ocular biometric diurnal rhythms in emmetropic and myopic adults. *Invest Ophthalmol Vis Sci*. 2018; 59: 5176–5187. 30372744 10.1167/iovs.18-25389 PMC6203176

[43] Chakraborty R, Read SA, Collins MJ. Diurnal variations in axial length, choroidal thickness, intraocular pressure, and ocular biometrics. *Invest Ophthalmol Vis Sci*. 2011; 52: 5121–5129. 21571673 10.1167/iovs.11-7364

[44] Stone RA, Pardue MT, Iuvone PM, Khurana TS. Pharmacology of myopia and potential role for intrinsic retinal circadian rhythms. *Exp Eye Res*. 2013; 114: 35–47. 23313151 10.1016/j.exer.2013.01.001 PMC3636148

[45] Kinane C, Calligaro H, Jandot A, et al. Dopamine modulates the retinal clock through melanopsin-dependent regulation of cholinergic waves during development. *BMC Biol*. 2023; 21: 146. 37365544 10.1186/s12915-023-01647-6 PMC10294308

[46] Brewer GJ, Torricelli JR, Evege EK, Price PJ. Optimized survival of hippocampal neurons in B27-supplemented Neurobasal, a new serum-free medium combination. *J Neurosci Res*. 1993; 35: 567–576. 8377226 10.1002/jnr.490350513

[47] Goyal V, DeVera C, Baba K, et al. Photoreceptor degeneration in homozygous male Per2(luc) mice during aging. *J Biol Rhythms*. 2021; 36: 137–145. 33135952 10.1177/0748730420965285 PMC8722430

[48] Ebihara S, Marks T, Hudson DJ, Menaker M. Genetic control of melatonin synthesis in the pineal gland of the mouse. *Science (New York, NY)*. 1986; 231: 491–493. 10.1126/science.3941912 3941912

[49] Kasahara T, Abe K, Mekada K, Yoshiki A, Kato T. Genetic variation of melatonin productivity in laboratory mice under domestication. *Proc Natl Acad Sci USA*. 2010; 107: 6412–6417. 20308563 10.1073/pnas.0914399107 PMC2851971

[50] Balazs I, Purrello M, Rocchi M, Rinaldi A, Siniscalco M. Is the gene for steroid sulfatase X-linked in man? An appraisal of data from humans, mice, and their hybrids. *Cytogenet Cell Genet*. 1982; 32: 251.

[51] Milićević N, Ait-Hmyed Hakkari O, Bagchi U, et al. Core circadian clock genes Per1 and Per2 regulate the rhythm in photoreceptor outer segment phagocytosis. *FASEB J*. 2021; 35: e21722. 34160105 10.1096/fj.202100293RR

[52] DeVera C, Dixon J, Chrenek MA, et al. The circadian clock in the retinal pigment epithelium controls the diurnal rhythm of phagocytic activity. *Int J Mol Sci*. 2022; 23: 5302. 35628111 10.3390/ijms23105302 PMC9141420

[53] Vargas JA, Finnemann SC. Differences in diurnal rhythm of rod outer segment renewal between 129T2/SvEmsJ and C57BL/6J mice. *Int J Mol Sci*. 2022; 23: 9466. 36012733 10.3390/ijms23169466 PMC9408929

[54] LaVail MM. Rod outer segment disk shedding in rat retina: relationship to cyclic lighting. *Science (New York, NY)*. 1976; 194: 1071–1074. 10.1126/science.982063 982063

[55] Possidente B, Hegmann JP, Carlson L, Elder B. Pigment mutations associated with altered circadian rhythms in mice. *Physiol Behav*. 1982; 28: 389–392. 7079353 10.1016/0031-9384(82)90129-9

[56] Lynch CJ. The so-called Swiss mouse. *Lab Anim Care*. 1969; 19: 214–220. 4240230

[57] Schwartz WJ, Zimmerman P. Circadian timekeeping in BALB/c and C57BL/6 inbred mouse strains. *J Neurosc*. 1990; 10: 3685–3694. 10.1523/JNEUROSCI.10-11-03685.1990 PMC6570095 2230953

[58] Meek KM, Knupp C. Corneal structure and transparency. *Prog Retin Eye Res*. 2015; 49: 1–16. 26145225 10.1016/j.preteyeres.2015.07.001 PMC4655862

[59] Ralph MR, Shi SQ, Johnson CH, et al. Targeted modification of the Per2 clock gene alters circadian function in mPer2luciferase (mPer2Luc) mice. *PLoS Comput Biol*. 2021; 17: e1008987. 34048425 10.1371/journal.pcbi.1008987 PMC8191895

