# Supplementary material for: Circadian Rhythms in Murine Ocular Tissues Including Sclera Are Affected by Neurobasal A Medium Preincubation and Mouse Strain, but Not Sex
Source: Invest Ophthalmol Vis Sci. 2025 Jun 20;66(6):62. doi: 10.1167/iovs.66.6.62 (PMC12184798; doi:10.1167/iovs.66.6.62)
Supplement: Supplement 1 [file iovs-66-6-62_s001.pdf]

Circadian Rhythms in Murine Ocular Tissues including Sclera are affected by Neurobasal A Medium Preincubation, Mouse Strain, but not Sex

Nemanja Milićević^[1], Cristina Sandu[2], Etienne Challet[2], Teemu O. Ihalainen[1], Soile Nymark[1], Marie-Paule Felder-Schmittbuhl*[2]

[1] Faculty of Medicine and Health Technology, Tampere University, Finland

[2] Centre National de la Recherche Scientifique, Université de Strasbourg, Institut des Neurosciences Cellulaires et Intégratives, 8 Allée du Général Rouvillois, F-67084 Strasbourg, France

Corresponding authors: ^ nemanja.milicevic@tuni.fi; * feldermp@inci-cnrs.unistra.fr

ORCID: Nemanja Milićević 0000-0002-8062-7270; Cristina Sandu 0000-0001-8836-5837; Etienne Challet 0000-0001-9416-9496; Teemu O. Ihalainen 0000-0003-4351-8697; Soile Nymark 0000-0002-9274-0123; Marie-Paule Felder-Schmittbuhl 0000-0003-3539-1243

Supplemental material

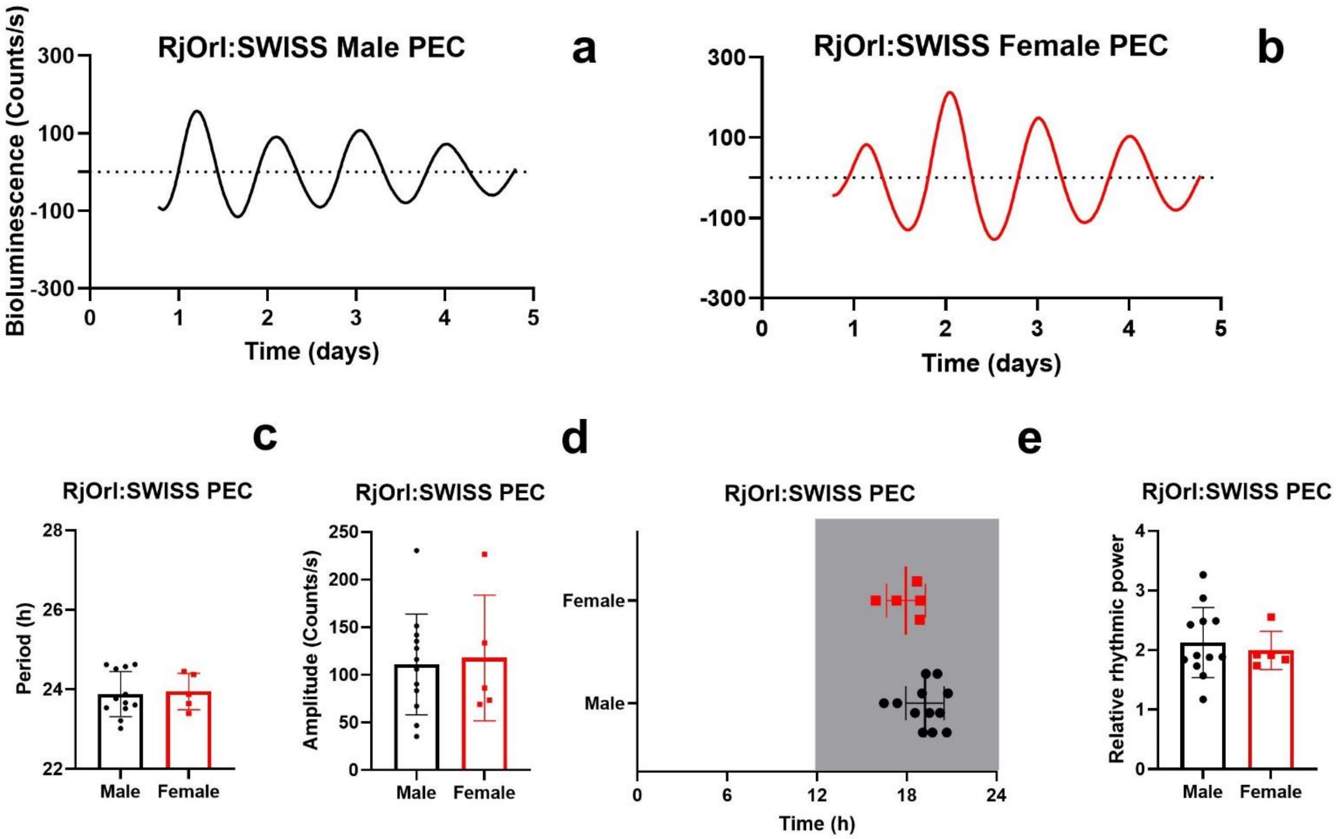

Figure S1. Sex does not affect circadian rhythms of PER2::LUC bioluminescence of PEC obtained from RjOrl:SWISS mice. Representative traces are shown of (a) male and (b) female PER2::LUC bioluminescence of PECs from RjOrl:SWISS mice. Animal sex did not significantly affect the (c) period length, (d) amplitude, (e) acrophase and (f) relative rhythmic power of PER2::LUC bioluminescence rhythms. Time in (e) is projected ZT with ZT12 = lights off. The gray rectangle represents subjective night-time. Individual data points are plotted together with means ± SD. PEC – posterior eye cup. N = 5 – 12
